# A MHz X-ray diffraction set-up for dynamic compression experiments in the diamond anvil cell

**DOI:** 10.1107/S1600577523003910

**Published:** 2023-06-15

**Authors:** Rachel J. Husband, Cornelius Strohm, Karen Appel, Orianna B. Ball, Richard Briggs, Johannes Buchen, Valerio Cerantola, Stella Chariton, Amy L. Coleman, Hyunchae Cynn, Dana Dattelbaum, Anand Dwivedi, Jon H. Eggert, Lars Ehm, William J. Evans, Konstantin Glazyrin, Alexander F. Goncharov, Heinz Graafsma, Alex Howard, Larissa Huston, Trevor M. Hutchinson, Huijeong Hwang, Sony Jacob, Johannes Kaa, Jaeyong Kim, Minseob Kim, Egor Koemets, Zuzana Konôpková, Falko Langenhorst, Torsten Laurus, Xinyang Li, Jona Mainberger, Hauke Marquardt, Emma E. McBride, Christopher McGuire, James D. McHardy, Malcolm I. McMahon, R. Stewart McWilliams, Alba S. J. Méndez, Anshuman Mondal, Guillaume Morard, Earl F. O’Bannon, Christoph Otzen, Charles M. Pépin, Vitali B. Prakapenka, Clemens Prescher, Thomas R. Preston, Ronald Redmer, Michael Roeper, Carmen Sanchez-Valle, Dean Smith, Raymond F. Smith, Daniel Sneed, Sergio Speziale, Tobias Spitzbart, Stephan Stern, Blake T. Sturtevant, Jolanta Sztuk-Dambietz, Peter Talkovski, Nenad Velisavljevic, Cara Vennari, Zhongyan Wu, Choong-Shik Yoo, Ulf Zastrau, Zsolt Jenei, Hanns-Peter Liermann

**Affiliations:** a Deutsches Elektronen-Synchrotron DESY, Notkestr. 85, 22607 Hamburg, Germany; b European XFEL, Holzkoppel 4, 22869 Schenefeld, Germany; c SUPA, School of Physics and Astronomy, and Centre for Science at Extreme Conditions, The University of Edinburgh, Peter Guthrie Tait Road, Edinburgh EH9 3FD, United Kingdom; d Lawrence Livermore National Laboratory, Physical and Life Science Directorate, Livermore, CA 94550, USA; e University of Oxford, Department of Earth Sciences, South Parks Road, Oxford OX1 3AN, United Kingdom; f The University of Chicago, Consortium for Advanced Radiation Sources, 5640 South Ellis Avenue Chicago, IL 60637, USA; g Los Alamos National Laboratory, Shock and Detonation Physics (M-9), PO 1663, Los Alamos, NM 87545, USA; h Department of Geosciences, 255 Earth and Space Sciences Building (ESS), Stony Brook, NY 11794-2100, USA; i Carnegie Science, Earth and Planets Laboratory, 5241 Broad Branch Road, NW, Washington, DC 20015, USA; j Washington State University, Department of Chemistry and Institute for Shock Physics, Pullman, WA 99164, USA; k Technische Universität Dortmund, Fakultät Physik/DELTA, Maria-Goeppert-Mayer-Straße 2, 44227 Dortmund, Germany; l Hanyang University, Department of Physics, 17 Haengdang Dong, Seongdong gu Seoul 133-791, Republic of Korea; m Institut für Geowissenschaften, Friedrich-Schiller-Universität Jena, Carl-Zeiss-Promenade 10, 07745 Jena, Germany; n SLAC National Accelerator Laboratory, 2575 Sand Hill Road, Menlo Park, CA 94025, USA; o Universität Münster, Institut für Mineralogie, Corrensstraße 24, 48149 Münster, Germany; p Univ. Grenoble Alpes, Univ. Savoie Mont Blanc, CNRS, IRD, Univ. Gustave Eiffel, ISTerre, 38000 Grenoble, France; q CEA, DAM, DIF, 91297 Arpajon Cedex, France; r Université Paris-Saclay, CEA, Laboratoire Matière en Conditions Extrêmes, 91680 Bruyères-le-Châtel, France; s Albert-Ludwigs University of Freiburg, Institute of Earth and Environmental Sciences, Hermann-Herder-Str. 5, D-79104 Freiburg, Germany; t Universität Rostock, Institut für Physik, Albert-Einstein-Straße 23–24, 18059 Rostock, Germany; u Argonne National Laboratory, High Pressure Collaborative Access Team (HPCAT), X-ray Science Division (XSD), 9700 S. Cass Avenue, Lemont, IL 60439, USA; v Deutsches GeoForschungsZentrum GFZ, Telegrafenberg, 14473 Potsdam, Germany; ESRF and Université Grenoble Alpes, France

**Keywords:** extreme conditions science, X-ray free-electron lasers, diamond anvil cells, dynamic compression

## Abstract

An experimental platform for dynamic diamond anvil cell (dDAC) research has been developed at the High Energy Density (HED) Instrument at the European X-ray Free Electron Laser (European XFEL). Advantage was taken of the high repetition rate of the European XFEL (up to 4.5 MHz) to collect pulse-resolved MHz X-ray diffraction data from samples as they are dynamically compressed at intermediate strain rates (≤10^3^ s^−1^), where up to 352 diffraction images can be collected from a single pulse train. The set-up employs piezo-driven dDACs capable of compressing samples in ≥340 µs, compatible with the maximum length of the pulse train (550 µs). Results from rapid compression experiments on a wide range of sample systems with different X-ray scattering powers are presented. A maximum compression rate of 87 TPa s^−1^ was observed during the fast compression of Au, while a strain rate of ∼1100 s^−1^ was achieved during the rapid compression of N_2_ at 23 TPa s^−1^.

## Abstract 2

A MHz X-ray diffraction set-up for the investigation of material behaviour under dynamic compression in a diamond anvil cell at intermediate strain rates has been developed at the High Energy Density (HED) instrument at the European XFEL.

## 1. Introduction

Many materials respond to extreme pressures in the gigapascal (GPa) to terapascal (TPa) regime by transforming to exotic new phases, where transitions may be accompanied by changes in electronic structure and/or chemical bonding (McMahon & Nelmes, 2006 ▸). High-pressure structural behaviour has traditionally been investigated using X-rays at static high pressures using a large volume press or diamond anvil cell (DAC) (McMahon, 2018 ▸), or under dynamic compression using gas guns or laser-driven compression (Wark *et al.*, 2022 ▸). These techniques are associated with very different timescales and strain rates ( 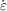 = dɛ/d*t*, where ɛ = Δ*V*/*V*), which can result in striking differences in determined phase diagrams (Gorman *et al.*, 2018 ▸; Pépin *et al.*, 2019 ▸; Coleman *et al.*, 2019 ▸; McBride *et al.*, 2019 ▸; Marshall *et al.*, 2021 ▸). Samples are compressed over minutes or hours in static compression experiments ( 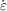 < 10^−3^ s^−1^), whereas the duration of shock and ramp compression experiments ( 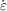 = 10^5^–10^9^ s^−1^) ranges from nanoseconds (laser-driven) to microseconds (gas gun-driven). Kinetic effects associated with phase transformations can sometimes result in significant differences in the observed phase transition pressures and temperatures under dynamic and static pressure conditions (Gorman *et al.*, 2018 ▸; Pépin *et al.*, 2019 ▸; Coleman *et al.*, 2019 ▸; Marshall *et al.*, 2021 ▸). In extreme cases, equilibrium phases can be completely absent in shock compression experiments (Gorman *et al.*, 2018 ▸; Pépin *et al.*, 2019 ▸), or metastable structures can form (Gorman *et al.*, 2018 ▸; Pépin *et al.*, 2019 ▸; Coleman *et al.*, 2019 ▸). Phase boundaries can also be influenced by the presence of significant shear stresses in shock and ramp compression experiments due to uniaxial compression (McBride *et al.*, 2019 ▸), the influence of which cannot be decoupled from kinetic effects.

Understanding kinetic effects is important to reconcile discrepancies between static and dynamic phase diagrams – particularly if material properties determined from dynamic compression studies are used to provide constraints on planetary interior models (Kraus *et al.*, 2017 ▸; Wicks *et al.*, 2018 ▸; Duffy & Smith, 2019 ▸; Coppari *et al.*, 2021 ▸; Kim *et al.*, 2022 ▸). However, despite the interest in rate- and timescale-related phenomena, material behaviour at intermediate compression rates ( 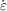 = 10^−3^–10^3^ s^−1^) remains relatively unexplored. This regime is accessible using dynamic DAC drivers (dDACs) (Evans *et al.*, 2007 ▸; Sinogeikin *et al.*, 2015 ▸; Jenei *et al.*, 2019 ▸; Yan *et al.*, 2022 ▸), which utilize piezoelectric actuators to compress samples in timescales ranging from milliseconds to several minutes. The maximum achievable strain rate is determined by the minimum compression timescale (*i.e.* piezo rise time) and largest accessible pressure range (*i.e.* force generation), where the latest generation of dDAC drivers can compress samples to the upper pressure limit of the diamond anvils in ∼1 ms. For example, strain rates up to 500 s^−1^ were generated during the fast compression of Au in 2.5 ms, which corresponds to the maximum compression rate (160 TPa s^−1^) reported to date (Jenei *et al.*, 2019 ▸). The dDAC also offers the additional advantage in that compression can either be performed hydro­statically through the use of a pressure-transmitting medium (PTM) or non-hydro­statically (without a PTM); the influence of the strain rate and stress state can therefore be decoupled.

Unlike conventional dynamic compression platforms where compression is inherently accompanied by high-temperature generation, dDAC compression does not necessarily lead to an increase in the sample temperature. The ability to compress along an isotherm means that dDAC experiments enable the exploration of well defined regions of pressure–temperature *(P–T*) space which are inaccessible using shock or ramp compression techniques, making it suitable for equation-of-state (EoS) measurements in which the *P–T* path must be well known. Although the temperatures generated in shockless ramp compression (*i.e.* quasi-isentropic) are low enough for solid phases to be studied beyond 1 TPa (Fratanduono *et al.*, 2020 ▸, 2021 ▸; Gorman *et al.*, 2023 ▸) – significantly cooler than the shock Hugoniot – compression is not isothermal, and temperature determination can be very challenging as temperatures are often too low to measure with conventional streaked pyrometry (Gregor *et al.*, 2016 ▸).

Investigation of material behaviour during dDAC compression requires appropriate time-resolved diagnostics which are compatible with the compression timescale. The development of high-frame-rate photon-counting X-ray detectors such as the LAMBDA (Pennicard *et al.*, 2013 ▸) and the EIGER has permitted time-resolved X-ray diffraction (XRD) studies of dDAC-compressed samples to be performed at synchrotron radiation sources (Marquardt *et al.*, 2018 ▸; Méndez *et al.*, 2021 ▸, 2022 ▸; Husband, O’Bannon *et al.*, 2021 ▸; Schoelmerich *et al.*, 2022 ▸; O’Bannon *et al.*, 2022 ▸), where high-*Z* sensors enable efficient detection of high-energy photons which are essential to penetrate the diamond anvils (≥10 keV) and provide sufficient access to reciprocal space for XRD experiments. However, minimum exposure times of ∼500 µs have in practice limited kinetic studies to compression rates of ∼1 TPa s^−1^ ( 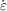 ≃ 10^−1^ s^−1^) – two orders of magnitude slower than achievable with the dDAC – in order for phase transition boundaries to be determined to within ∼0.25 GPa (Husband, O’Bannon *et al.*, 2021 ▸; O’Bannon *et al.*, 2022 ▸). Under faster compression rates, the change in the sample pressure within each detector exposure (*i.e.* the pressure resolution) is too large to accurately pinpoint phase boundaries. For example, the pressure resolution is limited to ∼40 GPa at the maximum reported compression rate (160 TPa s^−1^) with current detector technology, whereas 3 µs exposure times would be required for a resolution of ∼0.5 GPa. Although the next generation of photon-counting detectors promises about a tenfold increase in collection rate, this is still far from what is required to utilize the full dDAC capabilities. Synchrotron experiments are further limited by the available flux, where high-frame-rate data collection is limited to mid- or high-*Z* materials (Husband, O’Bannon *et al.*, 2021 ▸, 2022 ▸) as low-*Z* materials require much longer exposure times [*e.g.* ∼100 ms for H_2_O (Méndez *et al.*, 2021 ▸) and ∼1 s for Li (Frost *et al.*, 2019 ▸)].

The ability to collect meaningful data at strain rates above 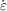 ≃ 10^−1^ s^−1^ requires access to faster X-ray diagnostics such as those available at the European XFEL. The unique time structure of the European XFEL, in which a series of intense, ultrashort-timescale (<50 fs) X-ray pulses are produced at repetition rates up to 4.5 MHz, offers the potential to study material behaviour in a ≤550 µs time window. This pulse train length is well suited to compression timescales achievable with current dDAC technology (≥340 µs), and the spacing between pulses (222 ns, 444 ns, 888 ns, *etc.*) provides the required time resolution to pinpoint phase boundaries within ∼0.1 GPa. The European XFEL bunch structure has previously been used to study DAC compressed samples under dynamic temperature conditions using the DAC platform in interaction chamber 2 (IC2) (Liermann *et al.*, 2021 ▸) at the High Energy Density (HED) instrument (Zastrau *et al.*, 2021 ▸), where sequential X-ray pulses were used to probe the high-temperature state induced by the previous pulse in a pump–probe fashion (Meza-Galvez *et al.*, 2020 ▸; Liermann *et al.*, 2021 ▸; Hwang *et al.*, 2021 ▸). However, these experiments did not have access to pulse-resolved XRD capabilities, but consecutive diffraction patterns of the pulse train were accumulated in a single image.

Here, we describe the integration of the latest generation of dDAC drivers into the DAC platform at the HED instrument. An Adaptive Gain Integrating Pixel Detector (AGIPD) (Allahgholi *et al.*, 2019 ▸) is used to collect pulse-resolved XRD data from dDAC compressed samples at the intra-train repetition rate of the European XFEL, providing time-resolved structural information during compression/decompression of the sample. Results from the fast compression of gold (Au) are presented to illustrate how X-ray heating can be minimized even in high-*Z* systems, which is important for kinetic studies where the *P–T* path must be well constrained. We also discuss the results from a series of experiments on four different phase-transforming materials – bis­muth (Bi), titanium (Ti), nitro­gen (N_2_) and water (H_2_O) – demonstrating the suitability of this platform for the study of a wide range of materials, from high- and mid-*Z* metallic systems to low-*Z* molecular solids. These data were collected as part of a DAC community proposal (#2592, by Liermann and Jenei, https://doi.org/10.22003/XFEL.EU-DATA-002592-00), and are examples of the first dDAC compression experiments performed at the European XFEL.

## 2. Experimental platform

The following section describes the integration of two different types of dDAC drivers (Section 2.1) in the DAC platform in IC2 at the HED instrument (Zastrau *et al.*, 2021 ▸; Liermann *et al.*, 2021 ▸). The set-up inside IC2 consists of three dDACs installed in a revolving sample exchanger alongside an optical microscope that is used for sample visualization and alignment (Section 2.2). Pulse-resolved XRD data are collected using an AGIPD detector positioned outside of IC2 behind an Al window (Section 2.3), and the relative timing between the dDAC compression and the XFEL pulse train is controlled by an electrical drive scheme (Section 2.4).

### 2.1. dDAC drivers

This set-up utilizes piezo-driven dDACs in which the sample pressure is increased by the force applied to the upstream side of a conventional DAC by a piezoelectric transducer. The force is controlled by the application of a time-dependent voltage waveform to the piezo, defining the temporal response of the pressure profile experienced during the experiment. The installation described here is compatible with two different dDAC designs developed at Deutsches Elektronen-Synchrotron (DESY) and Lawrence Livermore National Laboratory (LLNL) (Jenei *et al.*, 2019 ▸), specifically designed for high force generation (and consequently, high sample pressures) at short timescales. In both designs, the piezo and DAC are contained within an external metallic housing with a threaded cap to ensure good mechanical contact between the actuator and the DAC. The design of the metallic housing enables easy exchange of numerous pre-loaded, standard DACs with relatively few piezoelectric drivers; as such, they are suitable for experiments in which data will be collected from a large number of DACs. The angular aperture which is useable for X-ray diffraction is the same as for the standard DAC, which is dependent both on the DAC design and the diamond/seat combination used on the downstream side. The DESY dDAC is used in conjunction with symmetric, piston-cylinder type DACs offering a 69.8° X-ray opening angle when equipped with a standard diamond facing upstream and a Boehler Almax (BA) diamond with a 70° X-ray aperture facing downstream. The LLNL dDAC is compatible with LLNL-type DACs with a slotted opening proving 55/90° X-ray coverage on the downstream side (Jenei *et al.*, 2013 ▸). The LLNL DACs are equipped with standard cut diamonds mounted on a tungsten carbide (WC) seat facing upstream and a cubic boron nitride (cBN) seat facing downstream, so that X-ray coverage is limited by the slot opening in the DAC. The LLNL dDACs are equipped with an HPSt 1000/35-25/80 piezo actuator (Piezosystems Jena GmbH) and the DESY design is equipped with a 64-107 actuator (a PI Ceramic GmbH), both of which have a maximum operating voltage of 1000 V. When used in combination with the RCV 1000/7 amplifier (Piezosystems Jena GmbH), this gives a minimum rise time (0–1000 V) of 1.2 ms for the DESY dDAC and 340 µs for the LLNL dDAC. Full details of are given by Jenei *et al.* (2019 ▸).

**Figure 1:**
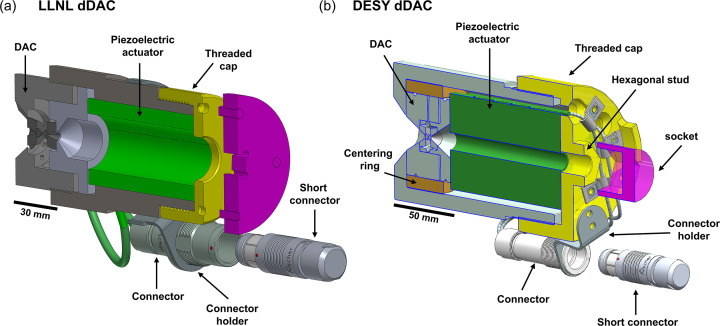
Schematics showing the dedicated (*a*) LLNL and (*b*) DESY dDAC housings. In (*a*), the threaded cap can be tightened with a torque wrench via an adapter which is attached to the dDAC housing. In (*b*), a torque wrench is coupled via a standard socket to the hexagonal stud. The high-voltage connector is mounted to the body of the dDAC by a connector holder. A short-circuit connector with a 10 kΩ discharge resistor is connected during transport and storage to avoid unwanted build-up of charge.

The cell response (the initiation of a systematic, measurable sample pressure variation versus applied voltage) depends on the pre-compression applied by the tightening of the threaded cap. Reproducible pre-compression is therefore important to ensure repeatable timing between the drive voltage and the XFEL probe. For this reason, the dDAC housings were modified from their original design to integrate an interface for a torque wrench. In the DESY design, this was achieved by machining a hexagonal stud interface to a size 24 mm socket directly into the cap, whereas for the LLNL design this was accomplished using a four-pin adapter which couples to four holes in the threaded cap and has a square interface in the middle of the plate to attach it to a torque wrench (Fig. 1 ▸).

### 2.2. Installation inside IC2

**Figure 2:**
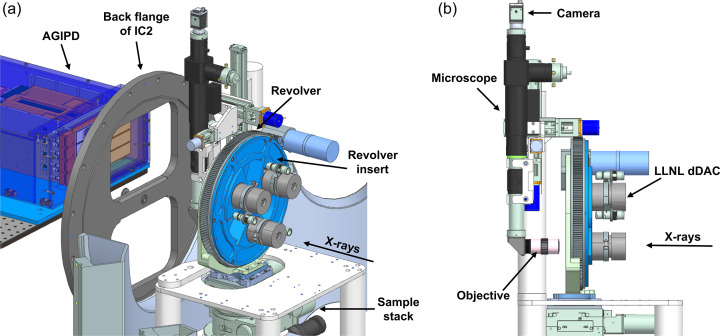
3D models showing two different viewpoints of the revolver and microscope installation inside IC2. Three LLNL-type dDACs are installed in the revolver insert. The microscope is shown in the X-ray beam path for viewing the sample.

The dDAC set-up is installed and operated under vacuum in IC2. In this set-up, three dDAC drivers are mounted in a motorized, carousel-type revolver which sits on a kinematic mount on the sample stack of the DAC platform (Liermann *et al.*, 2021 ▸) (Fig. 2 ▸). Switching between different dDACs is performed by rotating the revolver, which reduces the time required for sample exchange (including the time required to vent and pump IC2). The three dDACs are mounted in an interchangeable insert specific to the two different dDAC designs (DESY and LLNL), which can be exchanged without removing the entire revolver assembly. The inserts also feature three additional positions for reference and alignment samples, which typically consist of a diffraction standard for detector calibration (*e.g.* LaB_6_), a set of W round edges for focus characterization, and a YAG screen for visualization of the XFEL beam via X-ray fluorescence. When installed inside the chamber, the piezoelectric actuators of the three dDACs are connected to a high-voltage switchbox via individual hermetic feedthroughs mounted on a CF-type flange, which allows for the amplifier output to be switched between different actuators at the switchbox (Section 2.3).

Alignment of samples to the X-ray beam is performed using an optical microscope (Navitar) equipped with a vacuum-compatible camera (modified Basler), which is installed downstream of the revolver (Fig. 2 ▸). The microscope assembly is equipped with its own set of motorized translation stages which are used to align the camera to the X-ray beam, where the vertical translation stage additionally enables the objective to be removed from the beam path during data collection. Sample alignment parallel and perpendicular to the X-ray beam is performed using the *XYZ* translation stages of the sample stack.

### 2.3. AGIPD

A 500K AGIPD prototype (Allahgholi *et al.*, 2019 ▸) is used to collect pulse-resolved XRD images during compression. The AGIPD is capable of collecting up to 352 images at the maximum intra-train repetition rate (up to 4.5 MHz) and read out at the train repetition rate (10 Hz), allowing for XRD data to be collected from consecutive pulse trains. The AGIPD 500K consists of eight 128 × 512 pixel modules with Si sensors (500 µm thick) and a 200 µm × 200 µm pixel size which are arranged in a 2 × 4 (horizontal × vertical) configuration. Images collected when the pulse train is incident on the sample are subsequently extracted via the unique train ID. The AGIPD is positioned outside of IC2 on the detector bench, above the X-ray beam path. The detector bench allows for horizontal positioning of the AGIPD parallel and perpendicular to the X-ray beam. The back flange of IC2 is equipped with a 0.3 mm-thick Al window, which has ∼71% transmission at 18.1 keV.

### 2.4. Electrical drive scheme

**Figure 3:**
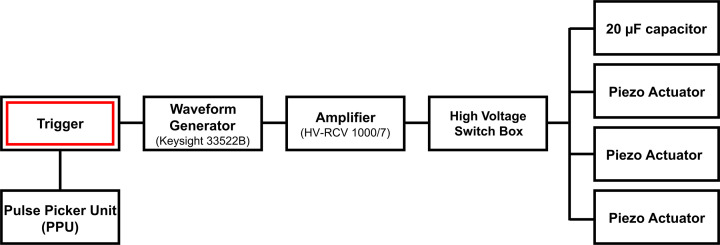
Electrical drive scheme of the dDAC driver.

The piezoelectric actuators are controlled by an electrical drive scheme (Fig. 3 ▸) consisting of a waveform generator (Keysight 33522B), a high-voltage amplifier (HV-RCV 1000/7) and a high-voltage switch box designed in-house at DESY. The switch box commutates the amplifier output between three different piezoelectric actuators and a 20 µF internal capacitor, and prevents switching at high voltage to avoid the piezoelectric actuators remaining charged after they are disconnected from the amplifier. Piezoelectric actuators are short-circuited via a 10 kΩ discharge resistor when disconnected from the amplifier to avoid unwanted build-up of charge during storage, handling, pre-compression and loading into the revolver insert.

European XFEL provides pulse trains at a 10 Hz repetition rate, where each pulse train has a duration of ≤550 µs and an intra-train pulse repetition rate of 4.5/2*n* MHz, where *n* is an integer (*i.e.* 2.25, 1.125, 0.75, 0.563 MHz,…). Synchronization of the drive voltage and the X-ray trains is achieved using the XFEL timing system, which provides 10 Hz trigger signals synchronized to the XFEL trains. A deterministic scheme is used to trigger the waveform generator and pulse picker unit (PPU) on a specific train ID, which allows for a single pulse train with a unique train ID to be transmitted to the sample. The trigger signal for the waveform generator has a local adjustable delay, so that data can be collected during different portions of the compression cycle. In the simplest mode of operation, timing of the voltage waveform is controlled by a single trigger signal (*i.e.* the waveform continues until completion) and data are collected using a single pulse train. Alternatively, the waveform can be defined in multiple segments which are triggered individually, so that data can be collected during several different sections of a compression cycle (*e.g.* during compression, hold and decompression). The experimental parameters can then be adjusted before the start of each individual segment such as the position of the sample in the XFEL beam (*e.g.* if the sample moved during compression), the X-ray intensity (*i.e.* beam attenuation) and the delay of the voltage waveform with respect to the start of the XFEL pulse train.

## 3. Dynamic compression experiments: experimental details

### 3.1. Overview

**Figure 4:**
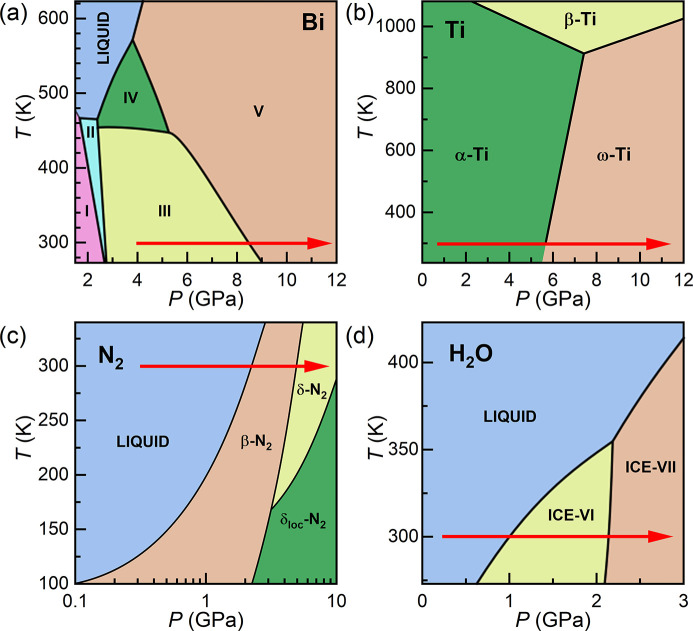
Phase diagrams of (*a*) Bi, (*b*) Ti, (*c*) N_2_ and (*d*) H_2_O, which are the phase transforming materials studied in this work. The red arrows show the ideal path taken during dDAC compression, highlighting the phase transitions targeted in these experiments. Phase diagrams are modified from the following references: Klement *et al.* (1963 ▸), Zhang *et al.* (2008*a* ▸), Bini *et al.* (2000 ▸), Pistorius *et al.* (1968 ▸).

The capabilities of this platform are illustrated by experiments on five different sample systems (Table 1 ▸). Results from experiments on Au are first presented to illustrate the approach taken to minimize sample heating during data collection. This is followed by results from compression experiments on four different phase transforming materials: Bi, Ti, N_2_ and H_2_O. Experiments were designed to target one or two different phase transitions in each material, which are highlighted in their respective phase diagrams in Fig. 4 ▸. For each sample, an internal diffraction calibrant was added to the sample chamber for independent pressure determination. Previous work has shown that internal diffraction standards can be used for dDAC experiments on samples loaded with a PTM, provided that the sample/pressure marker assembly remains hydro­static during compression (Husband, O’Bannon *et al.*, 2021 ▸). However, in most of the runs described here, diffraction from the calibrant was unfortunately not observed either because the diffracted signal was too weak or due to peak overlap with reflections from the sample or gasket.

Samples were screened at the Extreme Conditions Beamline (P02.2) (Liermann *et al.*, 2015 ▸) at the PETRA III synchrotron radiation source prior to the XFEL experiment to check the quality of the sample loading. Sample screening was performed before the DACs were loaded into the dDAC housing and a pre-torque was applied; consequently, the pressure determined during screening does not necessarily correspond to the starting pressure during the XFEL experiment.

### 3.2. XFEL parameters

The experiment was performed using a photon energy of 18.105 keV and an intra-train repetition rate of 1.1 MHz, which corresponds to an 888 ns spacing between XFEL pulses. The X-ray beam was focused using a series of compound refractive lenses to a focal spot size of ∼50 µm (h) × 50 µm (v), which was determined from edge scans using a polished W rod. XRD data were collected using the AGIPD at the intra-train repetition rate of 1.1 MHz, which was chosen to maximize the data collection window based on the 352-image limit of the AGIPD. The pulse train length was limited to 191 µs in this experiment, which provided a total of 216 pulses per train. The sample-to-detector distance (SDD) and orientation of the AGIPD were calibrated using an LaB_6_ NIST diffraction standard in conjunction with *DIOPTAS* software (Prescher & Prakapenka, 2015 ▸). The detector was positioned at an SDD of ∼480 mm, which provided a *Q*-range coverage of 2.15–3.85 Å^−1^. In this configuration, the *Q*-range coverage was limited by the size of the AGIPD.

The X-ray fluence was adjusted using a series of Si and diamond solid attenuators which determined the fraction of the XFEL beam which was transmitted to the sample (hereafter referred to as X-ray transmission and defined as a percentage of the unattenuated XFEL beam). The pulse energy was measured using a pulse-resolved intensity and position monitor (IPM) positioned upstream of the sample, which was cross-calibrated using an absolutely calibrated X-ray intensity gas monitor (XGM) before the start of the experiment. The pulse energy typically decreased over the course of the train, and the average pulse energy of each run is given in Table 1 ▸. All energies quoted in the remainder of the paper correspond to the energy at the sample position, corrected for absorption by the upstream diamond anvil using the attenuation length of diamond at 18.105 keV assuming ambient pressure density of diamond (Henke *et al.*, 1993 ▸).

### 3.3. Sample alignment

High-pressure beamlines at synchrotron light sources typically use absorption scans to align samples to the X-ray beam and position them at the centre of a rotation axis defining the SDD. Scanning procedures are more time consuming at XFEL sources for several reasons: long count times due to the pulsed nature of the source; the need for pulse-to-pulse normalization due to pulse energy variations between different trains; and the need to use a heavily attenuated beam to avoid damage from repeated XFEL irradiation. Absorption scanning is therefore typically reserved for essential alignment procedures where no other method is available.

Sample alignment was therefore performed using the optical microscope for the experiments described here. The microscope was aligned perpendicular to the X-ray beam based on the visual observation of X-ray induced fluorescence from a YAG screen at the sample position, after which the microscope was focused on the diffraction standard to define the sample plane (and SDD). Samples were then aligned to the focus of the optical microscope, which resulted in a ∼1 mm offset in the SDD due to the high refractive index of the diamond anvil which was accounted for in the detector calibration.

### 3.4. Compression details

The cells were pre-compressed in the dDAC housing by applying a 5.0 (2.5) N m torque to the DESY (LLNL) dDAC assemblies prior to compression. Samples were compressed/decompressed using a trapezoidal voltage waveform with the same rise, hold and fall times (Table 1 ▸) which was controlled by a single trigger, and data were collected during compression using a single ∼191 µs pulse train. The waveform was triggered before the start of the X-ray pulse train, and the delay time was chosen to target the pressure range of interest.

### 3.5. Diffraction data analysis

A total of 216 diffraction images were recorded for each run, which were radially integrated using *DIOPTAS* to produce 1D diffraction profiles from each frame. For runs performed using an X-ray transmission of >1%, the diffraction images were normalized to the IPM signal to account for intensity fluctuations of the incident beam energy during the pulse train prior to integration. This method of normalization was not possible for runs collected at lower X-ray transmissions because there was not sufficient signal on the IPM. The sample pressure was determined from the unit cell volume of the sample and/or pressure marker based on an EoS from the literature. With the exception of Bi-III, α-Ti and β-N_2_, unit cell volumes were calculated from the position of the most intense diffraction peak, as other peaks were typically too weak for the peak position to be accurately determined. Although two reflections were clearly observed in Au, the unit cell volume was determined from the (111) reflection because it is least affected by uniaxial stress (Takemura & Dewaele, 2008 ▸). The compression rate was determined based on a linear fit to the pressure–time data in a selected time window (Δ*t*) during which the pressure rise was approximately linear. The strain rate of the sample was calculated using 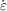 = (*V*_2_ − *V*_1_)/(*V*_1_Δ*t*), where *V*_1_ and *V*_2_ are the unit cell volumes of the sample at the start and end of the same time window (Δ*t*), respectively.

## 4. Results

### 4.1. X-ray heating evaluation

Accurate knowledge of the *P–T* path is important in dynamic compression studies to discriminate how pressure and temperature affect phase transformation kinetics. XFEL experiments can be complicated by X-ray heating due to the high energy density of pulses. Unwanted X-ray heating is particularly problematic for high-*Z* materials, as X-ray absorption is roughly proportional to *Z* ^4^ (Meza-Galvez *et al.*, 2020 ▸), which can potentially produce extreme temperatures in high-*Z* samples even when they are placed between conductive diamond anvils (Husband, McWilliams *et al.*, 2021 ▸). Although XFEL heating could potentially be used to perform dDAC studies at high temperatures, these experiments face the challenge of independent pressure and temperature determination. Furthermore, irradiation with sequential pulses from the pulse train does not provide uniform heating, but produces temperature gradients in the irradiated (and probed) area and a sawtooth-type temperature-time profile (Meza-Galvez *et al.*, 2020 ▸). For this reason, this work aimed to minimize heating in order to compress samples effectively isothermally room temperature.

X-ray heating can be minimized by reducing the total pulse energy, or by using a larger X-ray focal spot size to reduce the peak fluence. Increasing the beam size has the advantage that it minimizes the loss in diffracted intensity by increasing the illuminated sample volume (to the limit of the sample size), in contrast to higher attenuation which reduces the signal-to-noise ratio of the diffracted signal. A larger beam size also provides an improved powder average, which can be favourable for samples which show preferred orientation or larger crystallite sizes, especially due to the limited azimuthal coverage offered by the AGIPD. These experiments were therefore performed using an X-ray focal spot size of ∼50 µm FWHM, which was found to be a compromise between minimization of X-ray heating and parasitic diffraction and gasket heating originating from the tails of the beam. Internal pressure standards were chosen to have a smaller X-ray absorption coefficient (longer attenuation length, λ) than the sample to avoid unwanted heating of the pressure marker.

**Figure 5:**
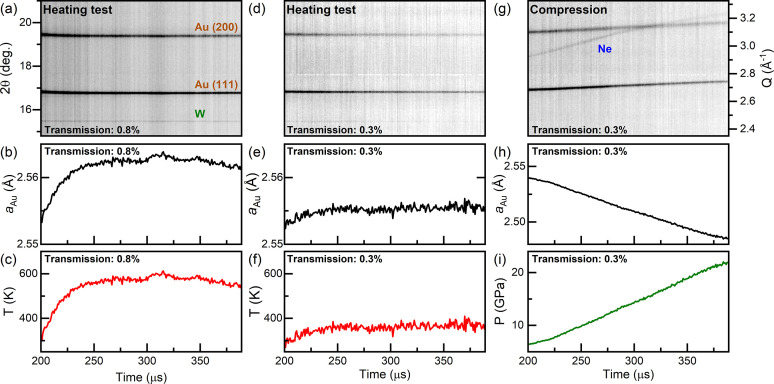
(*a*, *d*, *g*) Integrated XRD data collected from a sample of Au and Ne, illustrating the experimental procedure followed during a typical dDAC experiment. First, a heating test is performed at a given X-ray transmission (here 0.8%) to assess the extent of X-ray heating without compressing the sample [panels (*a*)–(*c*)]. If the temperature increase is too large, the heating test is repeated at a lower X-ray transmission [panels (*d*)–(*f*) at 0.3%] until the temperature rise is deemed acceptable for the experiment. Data are then collected using an appropriate X-ray transmission (here 0.3%) as the sample is compressed [panel (*g*)], and the pressure is determined based on the unit cell volume of the sample or pressure marker [panels (*h*) and (*i*)]. Pressure and temperature are determined assuming (*c*, *f*) isobaric and (*i*) isothermal conditions using the EoS of Au (Anderson *et al.*, 1989 ▸).

In order to evaluate the extent of X-ray heating to be expected during the dDAC experiment, the maximum allowable X-ray fluence for data collection was evaluated for each sample prior to the actual dynamic compression. This procedure is illustrated here for Au (Fig. 5 ▸), a high-*Z* element (*Z* = 79) that is highly absorbing with λ = 5.24 µm at 18.105 keV (Henke *et al.*, 1993 ▸). Au is of particular interest for DAC experiments because it is chemically inert, does not undergo any structural transitions in the pressure range accessible in DAC compression experiments, and has a well calibrated thermobaric EoS in this pressure range (Anderson *et al.*, 1989 ▸). For this reason, it is commonly used as an internal diffraction standard for pressure determination. Due to the differing sensitivities of X-ray heating (∼*Z* ^4^) and diffraction intensity (∼*Z* ^2^) to sample *Z*, Au represents a scenario for maximum heating in these experiments.

The Au sample was prepared in an LLNL-type DAC equipped with standard cut diamonds with 300 µm culets. Discs of 5 µm-thick Au foil and 3 µm-thick W foil were loaded into a stainless steel gasket with a 150 µm-diameter hole, and Ne was used as a PTM. XRD data collected at P02.2 indicated the initial pressure to be 0.9 GPa using the EoS of Au (Anderson *et al.*, 1989 ▸) and 1.2 GPa using the EoS of W (Dewaele *et al.*, 2004 ▸).

A series of 216 XRD images were collected at 1.1 MHz at a given X-ray transmission (here 0.8% = 2 ± 1 µJ) without compressing the sample to evaluate heating (Fig. 5 ▸). The starting pressure was estimated to be 3.3 GPa from the Au EoS, which is below the solidification pressure of Ne (Klotz *et al.*, 2009 ▸). The ∼2 GPa pressure increase from that measured at P02.2 is most likely due to the pre-torque applied to the DAC. The temperature increase was determined from the thermal expansion of the Au (Anderson *et al.*, 1989 ▸) assuming the pressure remained constant throughout the run. If sample heating of >50 K was detected, the test was successively repeated at a lower X-ray transmission until an appropriate transmission was found (here 0.3%, below the detection limit of the IPM of 0.5 µJ). Only then were XRD data collected during dynamic compression. In both of the examples described here (0.8 and 0.3% transmission), the width of the (111) Au reflection remained constant throughout the run, suggesting the absence of temperature gradients within the probed sample volume.

The Au sample was compressed using the LLNL dDAC with a 340 µs rise time up to 1000 V. XRD patterns were collected beginning 200 µs after the start of the voltage ramp, providing data coverage during the last 140 µs of the voltage rise and ∼50 µs of the plateau (Fig. 5 ▸). The pressure increase was limited to ∼3 GPa in the first 200 µs of the voltage ramp (before the start of XRD data collection), most likely related to a number of factors: the response of the dDAC assembly; the reproducibility of the amplifier response at such short rise times; and the volume drop associated with Ne solidification. Ne lines were visible in the initial XRD patterns collected during the ramp, confirming that crystallization had occurred during the first 200 µs of the voltage rise. The pressure increase was approximately linear across the 191 µs X-ray window with an average compression rate of 87 TPa s^−1^ ( 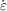 = 330 s^−1^), which was the highest compression rate achieved in this set of experiments. The 888 ns temporal resolution provided a pressure resolution of better than 0.1 GPa per frame at this compression rate, which is more than sufficient for kinetic studies.

### 4.2. Science case 1: dynamic compression of Bi

Bi is a high-*Z* (83), strongly scattering material which adopts numerous high-pressure polymorphs at relatively low *P–T* conditions. As such, it has been the focus of numerous laser-induced compression experiments which found significant deviations from the equilibrium phase diagram (Smith *et al.*, 2008 ▸; Gorman *et al.*, 2018 ▸; Pépin *et al.*, 2019 ▸). Previous dDAC studies also reported rate-dependent behaviour (Yang *et al.*, 2019 ▸; Husband, O’Bannon *et al.*, 2021 ▸), where the Bi-III/B-V phase boundary was observed to shift to higher pressures by as much as 2.5 GPa at ∼1 TPa s^−1^ (Husband, O’Bannon *et al.*, 2021 ▸). Studies under faster compression rates were limited both by the available flux at synchrotron radiation sources and the maximum effective detector frame rate (4 kHz), which prevented the accurate determination of phase transition boundaries under faster compression rates. The time resolution of the European XFEL therefore offers the opportunity to extend these studies under faster compression rates.

The Bi sample was prepared in an LLNL-type DAC equipped with diamonds with 300 µm culets. A ∼35 µm-diameter disk of 5 µm-thick Bi foil was loaded into a 30 µm-thick, 150 µm-diameter sample chamber in a stainless steel gasket, and a 5 µm-thick Cu disk was added as an internal diffraction standard. The Cu disk was spatially separated from the Bi foil to avoid unwanted chemical reactions that could be induced by X-ray heating. Ne was used as a PTM, and a small ruby sphere was added to the sample chamber for pressure determination after the loading process (Mao *et al.*, 1986 ▸). XRD data collected at P02.2 determined the pressure to be 1.5 GPa based on the unit cell volume of Cu (Dewaele *et al.*, 2004 ▸), and diffraction from the Bi foil showed the sample to be in the Bi-I phase (Degtyareva *et al.*, 2004 ▸).

A series of 216 XFEL diffraction images collected at 0.3% X-ray transmission (0.6 ± 0.4 µJ) prior to compression showed no evidence of heating in Cu, which determined a pressure of 4.3 GPa using the EoS from Dewaele *et al.* (2004 ▸). This pressure is lower than the Ne solidification pressure, and in the stability field of Bi-III. Although Bi-III has a large number of diffraction lines, only two were observed due to preferred orientation of the crystal grains. The spotty nature of the Bi-III diffraction patterns is consistent with observations in previous dDAC experiments on both powder and foil samples (Husband, O’Bannon *et al.*, 2021 ▸). Slight heating of Bi was observed in the first ∼25 µs, with the Bi-III host lattice expanding by ∼0.4%. The azimuthal intensity distribution of the Bi-III diffraction spots was observed to remain constant across all XRD images, ruling out the possibility that the sample had melted and recrystallized after probing and subsequent thermalization in the 888 ns between consecutive XFEL pulses.

**Figure 6:**
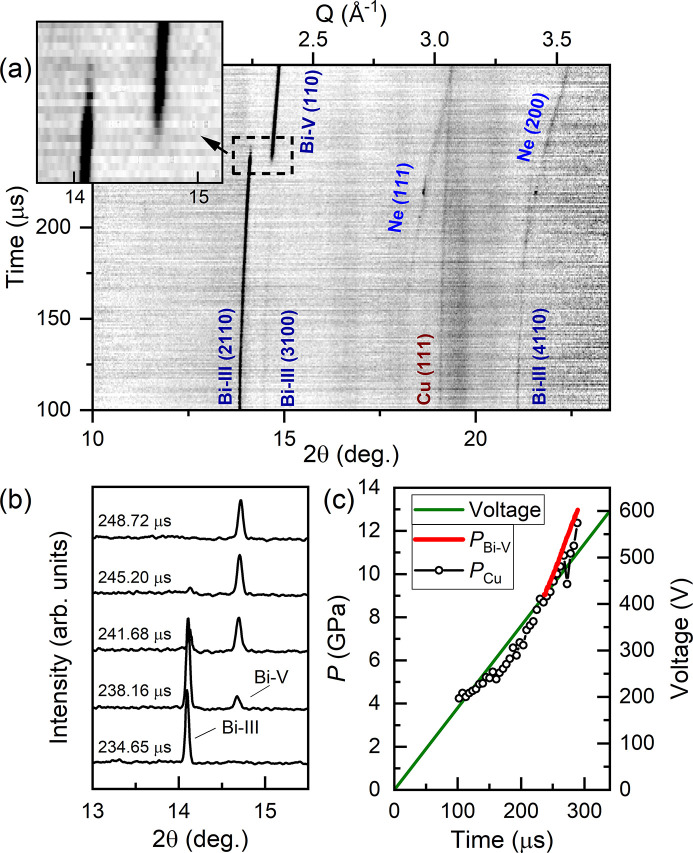
Dynamic compression of Bi. (*a*) Integrated XRD data as a function of time. (*b*) Selected integrated patterns illustrating the pressure-induced Bi-III/Bi-V structural phase transition. (*c*) Voltage applied to the piezo actuator and sample pressure as a function of time. In (*a*), the reflections from Bi-III, which has a host–guest structure, are identified using four Miller indices (*hklm*) (McMahon *et al.*, 2007 ▸). In (*c*), *P*_Bi_ was determined using the EoS of Bi-V (Degtyareva *et al.*, 2004 ▸) and *P*_Cu_ was determined using the EoS of Cu (Dewaele *et al.*, 2004 ▸). The intensity of the Cu (111) reflection was very weak, and so *P*_Cu_ was determined from the sum of six consecutive diffraction images.

Bi was compressed using the LLNL dDAC with a 340 µs rise time up to 600 V. The Bi-III/Bi-V phase transition was clearly observed in XRD data collected using 0.3% X-ray transmission (0.2 ± 0.4 µJ) (Fig. 6 ▸), where data collection started 100 µs after the start of the voltage waveform. The sample pressure was unchanged at the start of data collection, indicating a ∼100 µs pressure response time for the dDAC assembly. This slow response may be related to the solidification of Ne at ∼4.8 GPa. The pressure was therefore determined from the Cu (111) peak position in the sum of six consecutive diffraction images.

The number of observed Bi-III reflections was insufficient to determine the unit cell volume. However, a rough pressure estimate was obtained from a linear fit to the (2110) peak position based on lattice parameters reported in previous work (Degtyareva *et al.*, 2004 ▸), which determined a starting pressure of ∼4.3 GPa and a pressure of ∼8.3 GPa for the first observation of Bi-V. No evidence of the high-temperature Bi-IV phase was observed, suggesting that the temperature remained below ∼450 K (Klement *et al.*, 1963 ▸). The azimuthal position of the Bi-III reflections remained constant during compression, which again ruled out the possibility of melting and recrystallization between consecutive pulses.

Evidence of Bi-V was first observed 235 µs after the start of the voltage waveform, where the pressure was estimated to be 9.1 GPa based on the EoS of Bi-V (Degtyareva *et al.*, 2004 ▸). The azimuthal intensity distribution of Debye–Scherrer rings from Bi-V was much more uniform than for Bi-III, which is more favourable for identifying the onset of the phase transition. A total of 13 mixed-phase Bi-III/Bi-V diffraction patterns were observed [Fig. 6 ▸(*b*)], indicating a timescale of ≥11.4 µs for the transformation of the sample volume. The pressure increase was approximately linear in the Bi-V phase [Fig. 6 ▸(*c*)] with a compression rate of 76 TPa s^−1^ – two orders of magnitude faster than previous work (Husband, O’Bannon *et al.*, 2021 ▸) – which corresponds to a strain rate of 710 s^−1^ in Bi-V. The observed transition pressure is the same as previously observed for Bi foils compressed at significantly lower compression rates (∼10 GPa s^−1^) (Husband, O’Bannon *et al.*, 2021 ▸), which may be related to sample heating and the negative *P–T* slope of the Bi-III/Bi-V phase boundary.

### 4.3. Science case 2: dynamic compression of Ti

Ti (*Z* = 22) is an industrially and scientifically important metal due to its high strength-to-weight ratio and corrosion resistance. Ti undergoes a martensitic structural transition from the hexagonal close-packed α-Ti phase to the hexagonal ω-Ti phase on compression at room temperature, where the onset transition pressure is dependent on multiple factors including material purity (Hennig *et al.*, 2005 ▸), hydro­staticity (Errandonea *et al.*, 2005 ▸) and compression rate (Tomasino & Yoo, 2017 ▸). Each of these factors, or combinations of them, can lead to a variation in the transition pressure from ∼2 to 12 GPa. In particular, the presence of non-hydro­static stress shifts the phase boundary to lower pressures (Errandonea *et al.*, 2005 ▸), whereas compression rates ≥74 GPa s^−1^ shift it to higher pressures (Tomasino & Yoo, 2017 ▸). The transition is sluggish in all cases, and α- and ω-Ti coexist over a wide pressure range of 12 to ∼17 GPa. The experiment described here was focused on the influence of compression rate on the α–ω phase transition pressure in Ti loaded without a PTM. This highlights the unique feature of the dDAC, which is that the stress state of the sample can be varied through the use (or absence) of a PTM.

A high-purity Ti sample [impurities (by weight): 360 parts per million (p.p.m.) O, 60 p.p.m. C, 10 p.p.m. N, 14 p.p.m. H, 4 p.p.m. Al, 3 p.p.m. V and 5 p.p.m. Fe (Velisavljevic *et al.*, 2014 ▸)] was loaded into a symmetric DAC equipped with 300 µm culet diamonds. A stainless steel gasket with a 22 µm-thick, 150 µm-diameter gasket hole was used, and LiF and cBN were added to the sample chamber as internal pressure standards. Screening data collected at P02.2 confirmed that Ti was in the α-Ti phase, and the sample pressure was estimated to be 0.3 GPa based on cBN (Goncharov *et al.*, 2007 ▸) and 0 GPa based on Ti (Zhang *et al.*, 2008*b* ▸). No diffraction signal from LiF was observed.

A series of 216 XFEL diffraction images collected at 3% X-ray transmission (7 ± 3 µJ) prior to compression determined a pressure of 0.4 GPa from α-Ti. No LiF reflections were visible in the patterns but several intense, single-crystal-like reflections were observed from cBN, which corresponds to a pressure of 0.3 GPa (Goncharov *et al.*, 2007 ▸). The temperature increase during the run was estimated to be ∼400 K based on the thermal expansion of cBN (Goncharov *et al.*, 2007 ▸) and ∼220 K from Ti (Zhang *et al.*, 2008*b* ▸).

**Figure 7:**
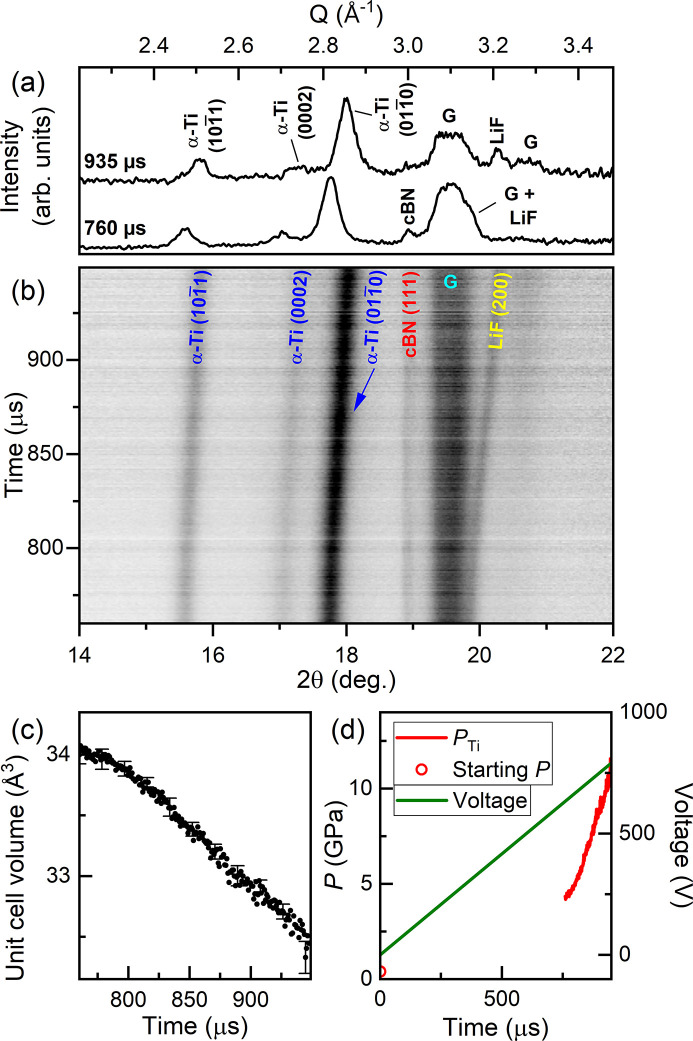
Dynamic compression of Ti. (*a*) Selected integrated diffraction patterns collected during the ramp, (*b*) integrated XRD data, (*c*) unit cell volume of α-Ti and (*d*) voltage applied to the piezo actuator and sample pressure as a function of time. In (*a*) and (*b*), **G** indicates reflections from the gasket. Error bars are shown for every 20th data point in (*c*).

The sample was compressed using the DESY dDAC with a 960 µs rise time up to 800 V, where XRD data were collected 760 µs after the start of the voltage waveform using 3% X-ray transmission (7 ± 3 µJ) (Fig. 7 ▸). All reflections could be identified as originating from α-Ti, LiF, cBN or the gasket. Unfortunately, none of the LiF or cBN reflections were observed across the entire dataset, highlighting a potential issue when low-*Z* pressure markers are used to avoid X-ray heating; the (110) LiF reflection was obscured by the (0002) α-Ti reflection in all patterns, the (200) reflection overlapped with one of the gasket reflections at the start of data collection, and the (111) cBN reflection became too weak to be clearly identified at the end of data collection [Fig. 7 ▸(*a*)]. The pressure was therefore estimated from the unit cell volume of α-Ti (Errandonea *et al.*, 2005 ▸). The sample pressure increased to 4.7 GPa in the first 760 µs (before the start of data collection), further increasing to 11.9 GPa at the end of the pulse train with an average compression rate of 37 TPa s^−1^ ( 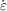 = 240 s^−1^ in α-Ti).

No evidence of ω-Ti was observed up to the highest pressure, which is >6 GPa above the α/ω equilibrium phase boundary at room temperature and >5.5 GPa at 520 K (Zhang *et al.*, 2008*a* ▸). An XRD map collected at the 16-ID-B beamline at HPCAT at the Advanced Photon Source in Argonne National Laboratory after the XFEL experiment found that the entire sample had fully transformed to ω-Ti and the pressure was estimated to be 21 GPa based on the ω-Ti EoS (Dewaele *et al.*, 2015 ▸), which provides a lower bound on the maximum pressure reached during the compression. The absence of ω-Ti at ∼12 GPa is surprising, as previous studies reported an onset transition pressure in samples loaded without a PTM ranging from 2–9 GPa [see Errandonea *et al.* (2005 ▸) and references therein]. However, the stress state of this sample is not well defined, as the limited 2θ coverage of the AGIPD prevents its determination using standard methods such as a line-shift analysis (Singh *et al.*, 1998 ▸). Further experiments are required to further constrain the extent of over-pressurization in Ti samples with a well defined stress state, shifting the X-ray window to higher pressures to also look at the phase coexistence region.

### 4.4. Science case 3: dynamic compression of N_2_

N_2_ exhibits a striking degree of polymorphism at high pressures, adopting seven different solid molecular phases (α, β, γ, δ, δ*, ɛ and ζ) below 100 GPa. These phases are characterized by triple-bonded N_2_ molecules and weak, intramolecular van der Waals bonding, with different degrees of lattice symmetry and orientational order. At higher pressures, structural changes are accompanied by changes in the chemical bonding such as transformations to amorphous (Gregoryanz *et al.*, 2001 ▸) and polymeric (Eremets *et al.*, 2004 ▸; Tomasino *et al.*, 2014 ▸) structures which are associated with a large hysteresis (Eremets *et al.*, 2001 ▸) and metastability (Gregoryanz *et al.*, 2001 ▸). The ability to collect time-resolved XRD of fast compressed N_2_ therefore offers the unique opportunity to study the structural evolution and phase transition kinetics in molecular solids at low pressures, and the compression-rate dependence of molecular to non-molecular transitions at higher pressures. The compression experiment described here focused on the low-pressure behaviour, specifically the pressure-induced solidification of N_2_.

The N_2_ sample was loaded into an LLNL-type DAC equipped with diamonds with 500 µm culets. The DAC was prepared using a stainless steel gasket with a 30–40 µm-thick, 150 µm-diameter sample chamber, and a small grain of LiF was added as an internal diffraction standard. A small ruby sphere was added to the sample chamber to determine the pressure after loading (Mao *et al.*, 1986 ▸). XRD data collected at P02.2 determined a pressure of 1.5 GPa based on the EoS of LiF (Dong *et al.*, 2014 ▸), and diffuse scattering from liquid N_2_ was clearly visible in the integrated diffraction patterns.

**Figure 8:**
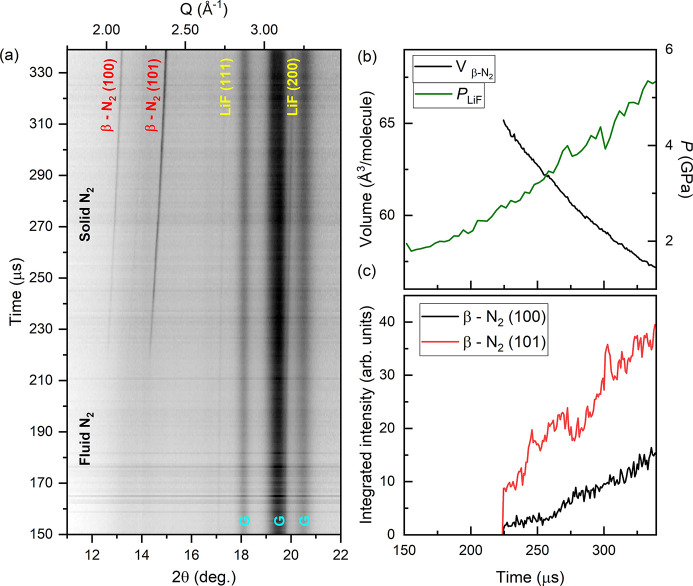
Dynamic compression of N_2_. (*a*) Integrated XRD data, (*b*) sample pressure and molecular volume of N_2_, and (*c*) integrated intensity of β-N_2_ reflections as a function of time. In (*a*), solidification of N_2_ can be observed at ∼220 µs, and **G** indicates gasket peaks.

A series of 216 XRD images collected at 75% X-ray transmission (220 ± 60 µJ) resulted in a <60 K temperature increase in LiF (Liu *et al.*, 2007 ▸), which was deemed acceptable for this experiment. The LiF reflections were very weak, and so their peak position was determined from the sum of four diffraction images. N_2_ was compressed in the LLNL dDAC using a 340 µs rise time to 1000 V, and XRD data were collected 150 µs after the start of the voltage waveform. Solidification of N_2_ was clearly observable in the XRD patterns [Fig. 8 ▸(*a*)], where β-N_2_ reflections were present from 223.92 µs until the end of the XRD series. The EoS of LiF determined a solidification pressure of 2.7 GPa, which is 0.3 GPa higher than that observed in static compression studies (Zinn *et al.*, 1987 ▸). An average compression rate of 20 TPa s^−1^ across the entire X-ray window was determined based on the EoS of LiF. The compression rate was 23 TPa s^−1^ in the region where β-N_2_ was observed, corresponding to a strain rate of 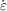 = 1100 s^−1^ in β-N_2_. N_2_ remained in the β-N_2_ phase up to at least 5.35 GPa, which is 0.35 GPa higher than the expected transition pressure to δ-N_2_ (Hanfland *et al.*, 1998 ▸). Further experiments are required to determine the extent of over-pressurization of the β-N_2_/δ-N_2_ transition.

### 4.5. Science case 4: dynamic compression of H_2_O

H_2_O has a complex phase diagram with 17 experimentally observed polymorphs, including two forms of amorphous ice at low temperatures and superionic ice at high temperatures (Millot *et al.*, 2018 ▸, 2019 ▸; Queyroux *et al.*, 2020 ▸; Prakapenka *et al.*, 2021 ▸; Weck *et al.*, 2022 ▸). On compression at room temperature, H_2_O crystallizes in the ice-VI structure at 1.05 GPa (Shimizu *et al.*, 1996 ▸), transforming to ice-VII at 2.1 GPa (Shimizu *et al.*, 1996 ▸). The ice-VI melting curve has a positive *P*–*T* slope, terminating at the liquid/ice-VI/ice-VII triple point at 2.2 GPa and 354.75 K (Pistorius *et al.*, 1968 ▸).

Laser-driven compression experiments reported striking deviations from the equilibrium phase diagram when H_2_O is compressed along the quasi-isentrope (Dolan *et al.*, 2007 ▸; Gleason *et al.*, 2017 ▸; Marshall *et al.*, 2021 ▸), which crosses the liquid/ice-VII phase boundary at 2.2 GPa (Myint *et al.*, 2017 ▸). Shockless ramp-compression experiments found that liquid H_2_O can be super-compressed to 8 GPa when compressed at 3 × 10^6^ TPa s^−1^ (Marshall *et al.*, 2021 ▸), well into the stability field of ice-VII. This is currently thought to represent the metastability limit of liquid H_2_O, where freezing is dominated by homogeneous nucleation (Marshall *et al.*, 2021 ▸). H_2_O has also been the focus of numerous dDAC studies which found that crystallization behaviour is highly dependent on compression rate: liquid H_2_O crystallizes directly in ice-VI on slow compression, but transforms directly to either ice-VII or amorphous ice on fast compression (Lee *et al.*, 2006 ▸; Chen & Yoo, 2011 ▸). Here, we focus on the pressure-induced crystallization of water at fast compression rates, providing direct structural identification using XRD which was not possible in earlier studies.

The H_2_O sample was loaded into a symmetric DAC equipped with 500 µm culet diamonds and a stainless steel gasket with a 39 µm-thick, 250 µm sample chamber. A small grain of cBN was added to the sample chamber as an internal pressure standard, which was chosen due to its low X-ray absorption [λ = 5889 µm (Henke *et al.*, 1993 ▸)] at this photon energy. A small ruby sphere was included for pressure determination after loading (Mao *et al.*, 1986 ▸). No ice reflections were observed in the XRD patterns collected at P02.2, and the presence of liquid H_2_O was confirmed by the observation of a diffuse scattering signal. The pressure was estimated to be 0 GPa based on the EoS of cBN (Goncharov *et al.*, 2007 ▸), which was slightly lower than the pressure of 0.27 GPa estimated from the ruby.

X-ray heating of H_2_O is a relatively minor issue because of its low X-ray absorption at 18.105 keV [λ = 7383 µm at 1 GPa (Henke *et al.*, 1993 ▸)], allowing for data to be collected with a relatively high X-ray fluence. No ice reflections were observed in the series of 216 diffraction images collected at 25% X-ray transmission (60 ± 20 µJ) prior to compression, suggesting that the sample was still in the liquid phase. This was confirmed by the observation of a weak diffuse scattering signal from liquid in the integrated diffraction patterns. Heating of H_2_O could not be assessed due to the absence of ice reflections. A pressure of 1.3 GPa was determined from cBN (Goncharov *et al.*, 2007 ▸), which is above the H_2_O solidification pressure. However, this could be related to the high bulk modulus of cBN, which makes it insensitive to small pressure changes.

**Figure 9:**
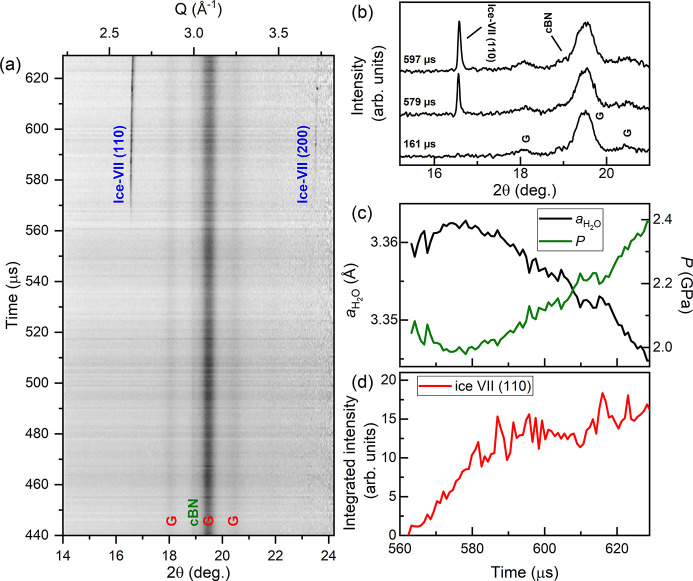
Dynamic compression of H_2_O. The panels show (*a*) integrated XRD data as a function of time, (*b*) selected integrated patterns showing the growth of the ice-VII reflection during compression, (*c*) the lattice parameter and corresponding pressure of ice-VII and (*d*) the integrated intensity of the (110) ice-VII reflection as a function of time. In (*c*), the pressure is determined using the EoS of ice-VII (Klotz *et al.*, 2017 ▸). In (*a*) and (*b*), **G** indicates reflections from the gasket.

The sample was compressed using an 840 µs rise time up to 700 V, and XRD data were collected 440 µs after the start of the voltage waveform using 25% X-ray transmission (60 ± 20 µJ) (Fig. 9 ▸). No ice reflections were observed during the first 125 µs of data collection, suggesting that H_2_O was still in the liquid phase. No evidence of ice-VI was observed during compression; instead, the sample transformed directly from liquid to ice-VII. Accurate determination of the pressure from cBN was prevented due to its partial overlap with the gasket peaks [Fig. 9 ▸(*b*)]. However, ice-VII indicated a crystallization pressure of 2.1 GPa (Klotz *et al.*, 2017 ▸), which is identical to the ice-VI/ice-VII equilibrium transition pressure at ambient temperature. The pressure remained approximately constant for ∼20 µs after the first observation of ice-VII [Fig. 9 ▸(*c*)], which is most likely related to the volume drop associated with the liquid/ice-VII transition and the time taken for the entire sample to transform. This is supported by the simultaneous increase in the integrated intensity of the (110) ice-VII reflection [Fig. 9 ▸(*d*)]. A linear pressure increase was observed after the pressure plateau, which corresponds to a compression rate of 8.1 TPa s^−1^ and a strain rate of 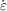 = 350 s^−1^ in ice-VII.

Our observation of the crystallization of ice-VII directly from super-compressed water is in good agreement with previous dDAC studies which used Raman and visual observation as phase identification methods (Lee *et al.*, 2006 ▸; Chen & Yoo, 2011 ▸), and is the first time in which this has been detected using XRD. However, we note that a temperature increase of just ∼50 K is sufficient to bypass the ice-VI phase on compression [Fig. 4 ▸(*d*)], which cannot be ruled out in this work. Further experiments are therefore required to decouple temperature- and rate-induced transition pathways in high-pressure H_2_O.

## 5. Outlook

The dDAC platform at the HED instrument was successfully commissioned as part of DAC community proposal #2592 (Liermann and Jenei), which demonstrated that this set-up can be used to collect time-resolved XRD data from a wide range of materials as they are dynamically compressed using piezo-driven dDACs. The ability to collect XRD data at the intra-train repetition rate of the European XFEL (1.1 MHz) provided a temporal resolution of 888 ns, which corresponds to a pressure resolution of <0.1 GPa per frame at the compression rates accessed in this study (up to 87 TPa s^−1^). This section describes future developments which will extend the capabilities of this set-up to a provide a more versatile platform.

Several upgrades are anticipated for the dDAC platform. A planned update of the high-voltage amplifier will provide higher currents up to 20 A, leading to shorter rise times and faster compression rates. This will be used in conjunction with the DESY dDAC, which has a rise time longer than the maximum pulse train length in the current configuration. In addition, IC2 was designed to accommodate a 1 megapixel (1M) AGIPD protruding into the vacuum chamber, which would significantly improve the *Q*-range and coverage due to the smaller SDD and increased detector area. Development is ongoing and implementation is planned for the near future.

There is also an ongoing development program of a high-*Z* version of the AGIPD 1M, which is designed for higher photon energies. From the facility side, European XFEL is capable of providing the HED instrument with photon energies up to 25 keV. Moving to higher photon energies offers two major advantages for dDAC experiments. First, X-ray heating is reduced because samples have a lower X-ray absorbance. Second, higher energies increase access to reciprocal space, which is fundamentally limited by photon energy due to the limited opening angle of the DAC. The combination of higher X-ray energies with larger detectors such as the AGIPD 1M has the potential to extend dDAC experiments to pair distribution function studies of liquid or amorphous materials.

The experiments described here were performed using a ∼200 µs pulse train; the standard configuration offered by European XFEL where the 600 µs pulse train is split between the three undulators (SASE 1–3). In this operation mode, the delay time between the XFEL and the dDAC voltage waveform must be carefully chosen to align the X-ray window with the pressure range of interest. European XFEL has recently implemented a ‘long pulse train’ mode in which every *n*th train is delivered only to SASE 2. This mode was commissioned with *n* = 100 and a 550 µs pulse train, where timing between the dDAC and the ‘long’ train can be performed using the methods described here. In this case, a total of 310 AGIPD images can be collected using a reduced intra-train repetition rate of 0.56 MHz, providing data coverage over the entire length of the pulse train.

## 6. Conclusion

This work describes an experimental set-up at the HED instrument at the European X-ray Free Electron Laser which is capable of collecting pulse-resolved XRD data from samples as they are dynamically compressed using piezo-driven diamond anvil cells. Compression timescales of ≥340 µs are perfectly suited to the pulse train length (200–550 µs), where the use of an AGIPD provides pulse-resolved XRD data at the intra-train repetition rate of the XFEL source. The capabilities of this platform are demonstrated by results from experiments on a range of different material systems with different X-ray absorption lengths. A maximum compression rate of ∼87 TPa s^−1^ was achieved during the fast compression of Au, and a maximum strain rate of ∼1100 s^−1^ was achieved during the compression of N_2_ at 18 TPa s^−1^. Varying the X-ray fluence and using a relatively large focal spot size limited X-ray heating even in the high-*Z* systems, demonstrating that this platform is suitable for kinetic studies in which the *P–T* path must be well constrained.

## Figures and Tables

**Table 1:** Details of the dynamic compression experiments described in this study The average pulse energy is corrected to account for absorption by the upstream diamond anvil using the attenuation length of diamond at 18.105 keV assuming the ambient pressure density of diamond (Henke *et al.*, 1993 ▸). The uncertainty in the average pulse energy corresponds to the standard deviation across the entire train.

	Sample details			X-ray parameters		Voltage waveform parameters			
Run No.	Sample	Pressure marker	PTM	X-ray transmission (%)	Energy/pulse on target (µJ)	dDAC assembly	Rise time (µs)	Maximum voltage (V)	Delay time (µs)
44	Au	W	Ne	0.3	0.2 ± 0.5	LLNL	340	1000	200
103	Bi	Cu	Ne	0.3	0.2 ± 0.4	LLNL	340	600	100
129	Ti	LiF, cBN	None	3	7 ± 3	DESY	960	800	760
140	N_2_	LiF	N/A	75	220 ± 50	LLNL	340	1000	150
93	H_2_O	cBN	N/A	25	60 ± 20	DESY	840	700	440
